# Mechanical Dispersion in Dilated and Non-Dilated Left Ventricular Cardiomyopathy: A New Frontier in Arrhythmic Risk Prediction

**DOI:** 10.3390/jcm15072687

**Published:** 2026-04-02

**Authors:** Nicoleta-Cosmina Hart-Foia, Alexandra Dădârlat-Pop, Renata Agoston, Florina Frîngu, Ioan-Alexandru Minciună, Carmen Cionca, Ruxandra Ștefana Beyer, Sebastian Onciul, Raluca Tomoaia, Dana Pop

**Affiliations:** 1 4th Department of Internal Medicine, Faculty of Medicine, “Iuliu Hațieganu” University of Medicine and Pharmacy, 400347 Cluj-Napoca, Romania; hada_nicoleta_cosmina@elearn.umfcluj.ro (N.-C.H.-F.); dadarlat.alexandra@yahoo.ro (A.D.-P.); florina.fringu@yahoo.com (F.F.); iaminciuna@gmail.com (I.-A.M.); pop67dana@gmail.com (D.P.); 2 Department of Cardiology, Rehabilitation Hospital, 400012 Cluj-Napoca, Romania; renata.agoston@yahoo.com; 3 Department of Cardiology, Heart Institute “N. Stăncioiu”, 400001 Cluj-Napoca, Romania; anda_bogdan@yahoo.com; 4 Medview Medical Center, 400913 Cluj-Napoca, Romania; carmen.cionca@gmail.com; 5 7th Department of Surgical Specialties, Faculty of Medicine, “Iuliu Hațieganu” University of Medicine and Pharmacy, 400347 Cluj-Napoca, Romania; 6 Faculty of Medicine, “Carol Davila” University of Medicine and Pharmacy, 020021 Bucharest, Romania; sebastian.onciul@gmail.com; 7 Department of Cardiology, Floreasca Emergency Clinical Hospital, 014461 Bucharest, Romania; 8 Leeds Institute of Cardiovascular and Metabolic Medicine, University of Leeds, Leeds LS2 9JT, UK

**Keywords:** mechanical dispersion, dilated cardiomyopathy, non-dilated left ventricular cardiomyopathy, ventricular arrhythmias, sudden cardiac death, myocardial fibrosis

## Abstract

**Background:** Sudden cardiac death (SCD) is a major challenge in dilated (DCM) and non-dilated left ventricular cardiomyopathy (NDLVC). Current management strategies, based on left ventricular ejection fraction (LVEF), the presence or extent of myocardial scar, and selected high-risk genetic variants, are insufficient to accurately identify patients at risk. Mechanical dispersion (MD), derived from speckle-tracking echocardiography, is a potential marker of arrhythmic risk that reflects variability in regional myocardial contraction timing. **Aim:** The purpose of this narrative review is to synthesize current evidence on the predictive role of MD for ventricular arrhythmias (VA) and SCD in DCM and NDLVC, with particular emphasis on its relationship to myocardial fibrosis (MF) and established echocardiographic markers. **Results:** Across prospective and retrospective cohorts of DCM patients, increased MD has consistently identified individuals at higher arrhythmic risk, often independently of LVEF and global longitudinal strain (GLS). Reported threshold values for risk prediction range from 50 ms to 90 ms, with hazard ratios confirming incremental prognostic accuracy. The relationship between MD and MF assessed by late gadolinium enhancement (LGE) on cardiac magnetic resonance (CMR) remains uncertain: some patients experience VA in the absence of LGE, while others display elevated MD despite no detectable focal MF, suggesting that additional mechanisms contribute to the arrhythmogenic substrate in DCM and NDLVC. **Conclusions:** MD may enhance SCD risk stratification in DCM and NDLVC by reflecting components of the arrhythmic substrate that are not detected by conventional markers.

## 1. Introduction

Dilated cardiomyopathy (DCM) is characterized by left ventricular (LV) dilation with or without right ventricular dilatation and systolic dysfunction [1], whereas non-dilated LV cardiomyopathy (NDLVC) can manifest as non-ischemic scarring or fatty replacement of the LV in the absence of dilatation with global or regional hypokinesia, or as isolated LV hypokinesia [2]. Both entities represent primary myocardial diseases not explained by loading conditions or coronary artery disease [1,2]. The global morbidity and mortality associated with these conditions remain high, primarily due to heart failure (HF) and malignant ventricular arrhythmias (VAs) leading to sudden cardiac death (SCD), despite advances in diagnosis and therapy [2]. SCD accounts for approximately 25–30% of all deaths in patients with DCM [3], raising the need for proper and early quantification of arrhythmogenic risk. The management of these patients is now increasingly directed based on LV ejection fraction (LVEF), presence or extent of scar, and the presence of genetic variants associated with higher arrhythmic risk [2]. SCD prevention using implantable cardioverter–defibrillators (ICDs) represents the standard of care in high-risk patients, yet identifying such patients remains a major clinical challenge [4].

## 2. Limitations of Validated Risk Markers

Currently, the indication for prophylactic ICDs in the primary prevention of SCD is guided mainly by reduced LV ejection fraction (LVEF ≤ 35%) [4]. However, LVEF alone has limited sensitivity and specificity for predicting arrhythmic events in DCM. While some patients with severely reduced LVEF may never develop malignant VAs, a significant proportion of those who experience SCD have a LVEF above the guideline threshold for ICD implantation [5], particularly within the NDLVC group [6].

Therefore, additional imaging markers have been validated. Global longitudinal strain (GLS), an echocardiographic marker derived from speckle-tracking echocardiography that quantifies myocardial deformation and detects subclinical systolic dysfunction, has been demonstrated to be an independent predictor of VAs in DCM patients [7]. Cardiac magnetic resonance (CMR) provides incremental value through both accurate LVEF assessment and tissue characterization. The presence and extent of myocardial fibrosis (MF) detected by late gadolinium enhancement (LGE) in cardiac magnetic resonance imaging (CMR) are strongly associated with an increased risk of SCD [8]. While LGE quantifies scar burden and GLS measures global contractile impairment, neither parameter reflects the temporal heterogeneity of myocardial contraction. This heterogeneity is directly responsible for promoting electrical dispersion and creating a substrate for re-entry circuits that facilitate malignant VAs [7]. Mechanical dispersion (MD), a strain-derived imaging marker reflecting the heterogeneity in the timing of regional contraction, overcomes these limitations. Elevated MD has been shown to predict arrhythmic events in various cardiovascular diseases, including DCM [9].

This review aims to synthesize current evidence on the role of MD as a predictor of ventricular arrhythmias and SCD in adult patients with DCM and NDLVC. The graphical abstract summarizes the overall concept.

Methodology—This study was conducted as a narrative review. An electronic literature search was conducted in the PubMed and Scopus databases over the last fifteen years, using combinations of key terms related to mechanical dispersion, dilated cardiomyopathy, non-dilated left ventricular cardiomyopathy and arrhythmic outcomes. Additional studies were identified by screening the reference lists of relevant publications. Original studies evaluating MD in relation to VAs or SCD in adult populations were included. Both prospective and retrospective observational studies were considered, including cohorts stratified by ischemic and non-ischemic DCM.

## 3. Concept of Mechanical Dispersion—Definition, Pathophysiological Link to Arrhythmogenesis and Technical Assessment

MD, defined as the standard deviation of time-to-peak systolic strain in 16 LV segments, is a strain-derived parameter that quantifies regional myocardial contraction. For a comprehensive understanding of its clinical relevance in SCD risk stratification, two key aspects need to be considered: first, the mechanisms linking MD to ventricular arrhythmogenesis; and second, the technical aspects of its measurement, including methodological strengths and limitations compared to other echocardiographic markers.

With regard to the first aspect, the pathophysiological link to ventricular arrhythmogenesis is centered around electromechanical heterogeneity, which constitutes the substrate for malignant VAs [10]. In DCM, several mechanisms are responsible for heterogeneity, although the underlying processes are not yet fully elucidated. An important driver is patchy mid-wall fibrosis, detectable by LGE on CMR [11]. Additional contributors include diffuse microscopic fibrosis [12,13] which is not detectable by LGE, altered gap–junction coupling with connexin-43 remodeling [14] and heterogeneous sympathetic denervation [15]. These abnormalities create areas of slow conduction and delayed activation, thereby promoting re-entry circuits, enhancing electrical dispersion and increasing susceptibility to triggered activity [10]. Notably, arrhythmic events occur even in patients without overt scar, indicating that electromechanical heterogeneity may exist independently of LGE [16]. Ultimately, these abnormalities manifest as mechanical asynchrony, which is captured by increased MD, thereby supporting its role as a valuable non-invasive marker of the arrhythmogenic substrate in DCM [7].

**Figure 1:**
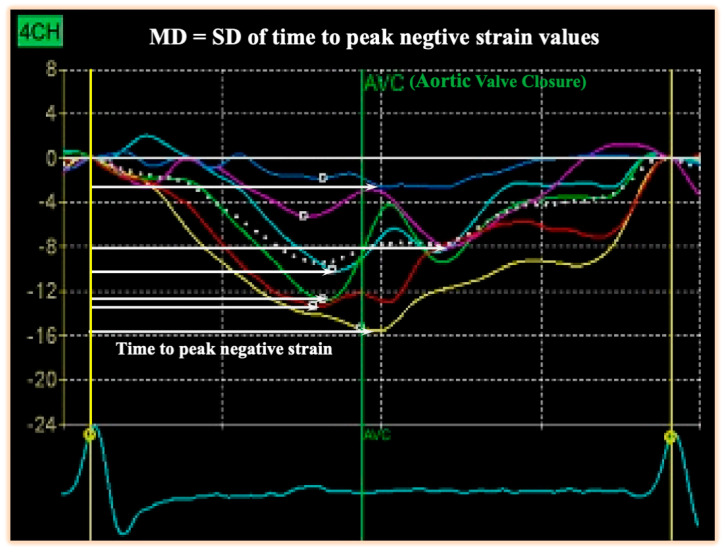
**Measurement of MD by speckle-tracking echocardiography in a DCM patient.** Strain curves from an apical four-chamber view are shown, each color representing one myocardial segment. Time to peak negative strain (white arrows) is measured from the onset of the Q/R complex on the surface ECG (yellow vertical line) to peak negative strain, including post systolic peaks when present. MD is calculated as standard deviation of these time-to-peak values across all 16 LV segments.

To enable accurate clinical use, the technical aspects of MD measurement need to be addressed. First, apical four-, three- and two-chamber views are acquired at adequate frame rates (50–90 frames/s) with simultaneous electrocardiogram (ECG) recording, ensuring compatibility with speckle-tracking software for subsequent analysis. Second, for each individual segment, the time from the onset of the Q/R complex on the surface ECG to that segment’s peak negative longitudinal strain is determined, including any post systolic peaks when present. Finally, MD is calculated as standard deviation of these time-to-peak values across all segments [7,9,17], as illustrated in Figure 1.

**Figure 2:**
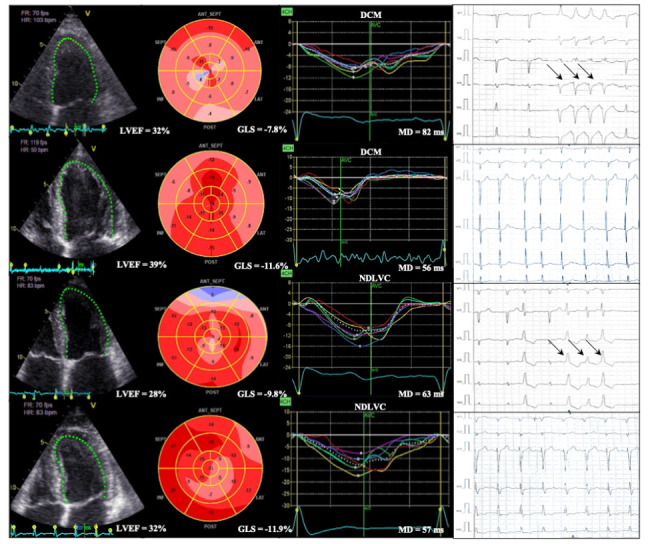
**MD, GLS, LVEF and corresponding electrocardiographic (ECG) recordings in representative patients with DCM and NDLVC, stratified by arrhythmic outcome.** Panels illustrate increased MD in patients with documented VA (black arrows), compared to patients without events.

By reflecting the temporal heterogeneity of myocardial contraction, MD provides a distinct and complementary approach to SCD risk prediction. Unlike conventional echocardiographic parameters such as LVEF and GLS, which primarily assess global or regional contractile function, MD captures mechanical dyssynchrony [18]. Moreover, given the complex mechanisms underlying the arrhythmogenic substrate in DCM, such abnormalities may be present even in patients with only subtle systolic dysfunction, suggesting that MD may help detect subclinical arrhythmic risk [7,19]. Clinical use of MD is influenced by image quality and conditions such as left bundle branch block (LBBB) or cardiac resynchronization therapy, and while standardized cut-off values in HF are not yet established, as is also the case for GLS, MD could still offer additional advantages in this population [7,9]. The technical strengths and limitations of MD, compared with LVEF and GLS—echocardiographic parameters validated for risk assessment in DCM, are summarized in Table 1. Examples are depicted in Figure 2.

## 4. Predictive Value of Mechanical Dispersion

### 4.1. Evidence Linking MD to Arrhythmic Outcomes and Proposed Threshold Values

In non-ischemic DCM, increased MD has consistently identified patients at higher arrhythmic risk, even in those with preserved or mildly reduced systolic function. Even though studies of MD have been heterogenous in terms of design, population and endpoints, data focusing on non-ischemic DCM suggest that threshold values to predict higher arrhythmic risk range from 50 ms to 72 ms in smaller cohorts, with higher values (90 ms) reported in larger or mixed cohorts, highlighting both the potential utility of MD, as well as the need for further validation together with conventional measures such as LVEF and strain [7,22,23,24,25]. Building on these results, the investigation was extended to patients with familial DCM, specifically lamin A/C (LMNA) mutation carriers, who are known to carry a particularly high arrhythmic risk. In this cohort, MD was able to identify arrhythmic risk even in the early stages of disease, when ventricular dimensions were normal or only mildly abnormal, indicating that MD and genetic information could be integrated to stratify arrhythmic risk in this high-risk population [19]. It would also be of interest to test this approach in carriers of other pathogenic variants, or even variants of uncertain significance, to better define risk in patients who are otherwise challenging to stratify.

The reported cut-off values and specific study design for predicting arrhythmic events are illustrated in Table 2.

### 4.2. Prognostic Accuracy Compared with Validated Echocardiographic Markers 

Even though its predictive role in identifying DCM patients at risk of VAs and SCD has been demonstrated, the extent to which MD provides incremental prognostic value beyond conventional markers remains to be fully elucidated. In the study by Haugaa et al. (2012) [7], MD remained a significant predictor of VAs in non-ischemic DCM, independent of LVEF or GLS. Similar findings were later confirmed in larger prospective and retrospective cohorts, where MD consistently demonstrated prognostic accuracy beyond LVEF or GLS [22,23]. In the current era of HF therapy, in which a substantial number of patients show improvement in LVEF and GLS under guideline-directed medical therapy [26], the predictive value of conventional measures such as LVEF and GLS may be attenuated, highlighting the potential of MD to identify residual electromechanical abnormalities and arrhythmic risk [23]. Finally, in a familial DCM cohort, MD was able to predict VAs in the early stages of the disease, when ventricular function was relatively preserved, whereas LVEF and GLS did not differ between patients with and without arrhythmic events [19]. This suggests that in genetic cardiomyopathies, where VAs can occur despite preserved ventricular function, MD may reveal subtle abnormalities and provide additional risk stratification. Overall, myocardial heterogeneity appears to play an important role in ventricular arrhythmogenesis, highlighting mechanical dyssynchrony preceding structural remodeling, a feature not detectable by conventional parameters.

### 4.3. Correlation Between Mechanical Dispersion and Myocardial Fibrosis

**Figure 3:**
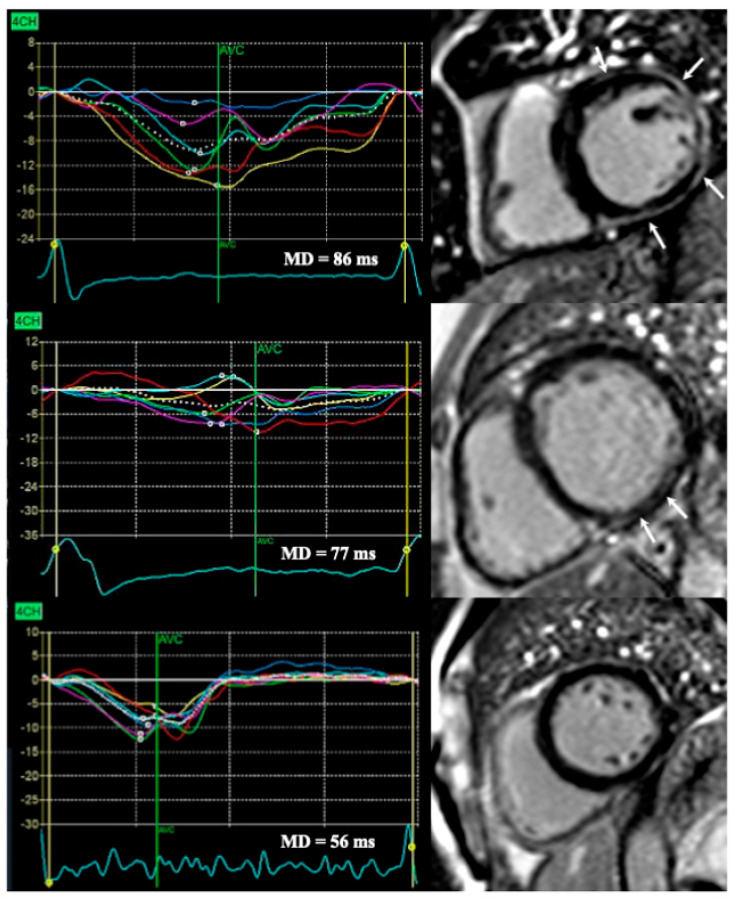
**Relationship between MD by echocardiography and focal MF by cardiac magnetic resonance in representative DCM patients.** The first case shows a patient with extensive MF (white arrows), the second one with limited MF (white arrows), and the third one without MF, each presented together with corresponding MD.

In terms of MF, both its presence and extent have been consistently linked to the prediction of future arrhythmic events in DCM [8]. In a cohort of mixed NICM patients with LVEF ≤ 35%, Gutman et al. (2020) [27] reported no correlation between MD and the presence or extent of LGE. In that study, LGE, but not MD, predicted mortality and malignant arrhythmic events, although the studied population was heterogenous and analysis of these findings in the DCM subgroup was not performed [27]. The presence of MF alters contraction in the affected segments [11], yet whether its extent defined by the number of segments involved, truly translates into greater electromechanical heterogeneity and higher arrhythmic risk remains elusive. Figure 3. exemplifies representative DCM patients with extensive, limited and absent MF on CMR together with their corresponding MD.

**Figure 4:**
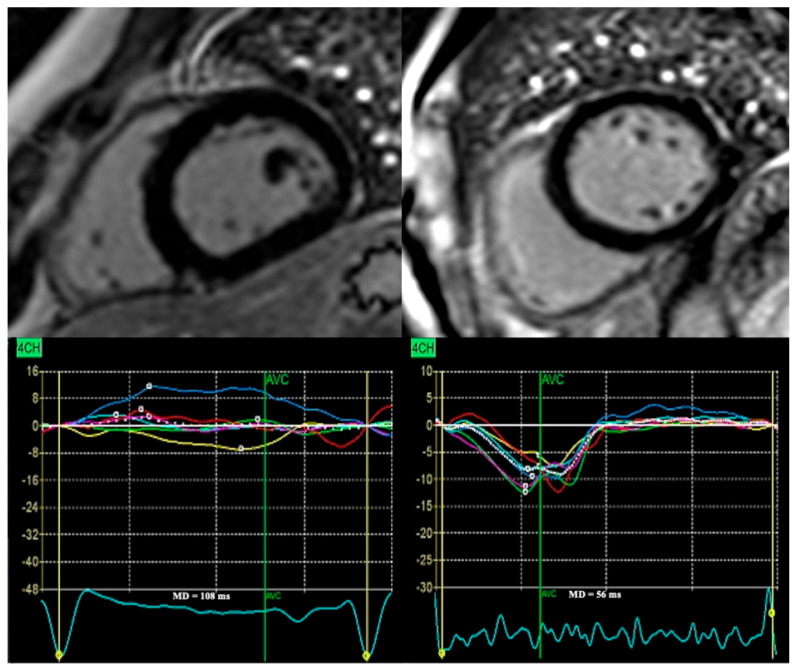
**MD in DCM patients without focal MF by cardiac magnetic resonance.** Despite the absence of MF, one patient presented with increased MD, whereas the other had low MD (<70 ms).

Importantly, some patients experience VAs without LGE [8], indicating that mechanical dyssynchrony may originate from mechanisms beyond MF and reflecting that arrhythmic substrates are not yet fully understood. This concept is illustrated in Figure 4, which shows two DCM patients without focal MF by CMR but with divergent MD values. Whether a relationship between MD and MF exists in DCM and NDLVC remains to be elucidated, and future studies are required to clarify whether MD and LGE provide overlapping, complementary or distinct information in these populations. By reflecting a broader pathophysiological spectrum including inflammation, myocyte disarray, diffuse interstitial fibrosis and electromechanical uncoupling, MD may play a major role in identifying high-risk patients [12,13,14,15]. Unlike CMR, MD is fast and widely available and can be easily implemented in routine echocardiographic practice, making it a valuable tool for risk stratification in DCM.

### 4.4. Sudden Cardiac Death Prediction in Genetic DCM

Genetic cardiomyopathies, present some of the greatest clinical challenges, as patients experience VAs and SCD early, often before LV dilatation or systolic dysfunction [2]. In a small cohort of LMNA carriers, Haugaa et al. (2015) showed that MD was independently associated with arrhythmic events [19]. These findings highlight the need for additional markers to better identify arrhythmic risk in the presence of subtle myocardial abnormalities that predispose to VAs and highlight the importance of further investigation in other genetic cardiomyopathies to determine whether MD can similarly improve risk stratification across these high-risk populations.

## 5. Additive Value, Limitations and Future Directions

### 5.1. Additive Value

When assessing arrhythmic risk in patients with DCM and NDLVC, an integrated approach is required. Electrical alterations can be captured using electrocardiographic markers associated with VAs, including repolarization abnormalities (e.g., QT interval), ventricular ectopy (e.g., premature ventricular complexes) [28], as well as measures of electrical heterogeneity such as heart rate variability parameters [29]. The structural substrate is best characterized by CMR through the assessment of myocardial fibrosis and scar burden using LGE, whereas the temporal heterogeneity of myocardial contraction is specifically reflected by MD.

Accordingly, MD is a promising marker of arrhythmic events in DCM and NDLVC, providing prognostic information beyond LVEF, GLS and N-terminal pro-B-type natriuretic peptide (NT-proBNP) [30]. It constitutes a valuable marker for identifying high-risk patients, particularly those with preserved or mildly reduced LVEF, in whom conventional criteria often fail to detect latent arrhythmic risk. By refining ICD selection strategies, MD may in the future help overcome the limitations of the traditional LVEF threshold. Considering that guideline-directed medical therapy (GDMT) promotes improvement and even recovery of systolic function, mainly [26] through enhanced contractility and reverse remodeling, it is clinically essential to investigate whether these benefits also translate into reduced electromechanical heterogeneity. Tracking MD dynamics in parallel with treatment response could open new pathways to link pharmacological therapy to arrhythmic risk modulation.

MD represents an accessible, non-invasive and easily repeatable echocardiographic parameter that can be obtained from standard speckle-tracking echocardiography without the need for additional acquisitions, supporting its integration into routine clinical evaluation of patients with DCM and NDLVC. However, the variability of reported MD cut-off values limits the use of fixed thresholds in clinical decision-making, requiring its interpretation within a multimodal framework.

### 5.2. Limitations

Despite its promising role, the current evidence on MD in DCM and NDLVC remains limited. Most available data are derived from relatively small, single-center observational cohorts—including both prospective and retrospective studies—with considerable heterogeneity in inclusion criteria, endpoints, and follow-up duration. Only a limited number of studies have enrolled exclusively DCM populations, further restricting the generalizability of the findings, while data specifically addressing NDLVC are particularly scarce. Taken together, these limitations indicate that the current evidence remains limited and largely based on observational studies, corresponding to a low level of evidence, with a lack of large prospective multicenter or randomized data.

In addition, several important gaps in the current literature should be acknowledged. The combined evaluation of MF by CMR and MD by speckle-tracking echocardiography for SCD risk assessment in DCM cohorts has not yet been systematically assessed. The predictive value of MD has not been fully validated in specific genetic subtypes of DCM, except for LMNA carriers. Furthermore, MD measurement is influenced by the need for high quality echocardiographic imaging and by variability across software vendors, although this limitation was less evident in current studies, as most used the same platform (GE). Finally, universally accepted cut-off values for predicting arrhythmic events in DCM patients are still lacking, hindering their use in routine clinical practice.

### 5.3. Future Directions

To address these limitations, large, long-term studies are needed to validate MD as a reliable marker for SCD risk assessment across the DCM spectrum. Multicenter prospective trials are necessary to investigate the role of advanced multimodality imaging in arrhythmic risk prediction, integrating MD, GLS, LVEF and LGE, to determine not only which marker is most reliable, but also whether these modalities provide complementary information on SCD risk prediction. A key focus should be on the role of MD in genotype-specific risk stratification, as this may be particularly relevant for carriers of high-risk genes.

## 6. Conclusions

MD may capture aspects of the arrhythmic substrate in DCM and NDLVC not detected by conventional markers, offering promise for more precise and clinically relevant SCD risk stratification.

## Figures and Tables

**Table 1:** Echocardiographic markers used for arrhythmic risk assessment in DCM: technical strengths and limitations [7,9,20,21].

Echocardiographic Marker	Strengths	Limitations
**2D LVEF**	- Universally available	- Operator and geometry-dependent
	- Low cost	- High intra-/inter-observer variability
		- Requires adequate imagine quality - Load-dependent (preload/afterload) - Insensitive to regional abnormalities - Not relevant in the context of NDLVC
**GLS**	- Angle-independent	- Vendor and software variability
	- Less operator-dependent - Faster image acquisition - Higher reproducibility than LVEF - More sensitive than LVEF for early systolic dysfunction	- Load-dependent - Requires adequate imagine quality, frame rate and ECG alignment - Reflects global myocardial contraction - Does not capture heterogeneity of contraction timing - Lack of standardized cut-offs across pathologies
**MD**	- Quantifies temporal heterogeneity of myocardial contraction - High sensitivity for detecting regional abnormalities - Less afterload dependency - Can reveal early dyssinchrony before structural remodeling or systolic dysfunction occurs [19] - Can provide prognostic information even in patients with NDLVC [19]	- Vendor and software variability - Requires adequate imagine quality frame rate and ECG alignment - Sensitive to tracking algorithms - Values may be artificially increased in the presence of LBBB or during resynchronization therapy - Lack of standardized cut-offs across pathologies

**Table 2:** Reported MD cut-offs for predicting arrhythmic events in cohorts including DCM patients.

Study (Year)	Study Design and Population	Endpoints	MD Threshold (ms) and Statistical Significance
Haugaa et al., 2012 [7]	Prospective observational cohort; 94 patients with non-ischemic DCM	Composite of SCD, documented sustained ventricular tachycardia (VT), appropriate therapy from a primary preventive ICD, syncope of cardiac cause	Predictor of arrhythmic events independent of LVEF (HR, 1.3; 95% CI, 1.1–1.5; *p* = 0.001). Cut-off > 72 ms; AUC 0.80, sensitivity 67%, specificity 89%
Haugaa et al., 2015 [19]	Prospective observational cohort; 33 LMNA mutation carriers (Familial DCM)	Nonsustained VT, sustained VT, ventricular fibrillation (VF)	MD 49 ± 14 ms vs. 38 ± 10 ms in event-free (*p* = 0.02). MD discriminated arrhythmic events (AUC 0.74, 95% CI 0.55–0.93), outperforming LVEF (AUC 0.64) and GLS (AUC 0.54)
Kosiuk et al., 2015 [24]	Case–control cohort; 20 NICM patients	Documented sustained VT and VF	MD > 50 ms, associated with twelve times higher risk of VT (OR 12.5, 95% CI 1.1–143.4, *p* = 0.024)
Matsuzoe et al., 2016 [25]	Retrospective cohort: 72 patients with HF with LVEF ≥ 35% who underwent ICD implantation, including 9 DCM patients	Appropriate ICD therapy for VT and/or VF	MD ≥ 101 ms, predictive of fatal VAs (AUC 0.685, *p* = 0.004). No specific cut-off in DCM subgroup
Melichova et al., 2021 [22]	Multicenter prospective observational cohort; 290 patients with ischemic and non-ischemic DCM (83 patients with non-ischemic DCM)	Composite of SCD, appropriate therapy from a primary preventive ICD and sustained VT	MD > 70 ms, predictive of life- threatening VAs (combined endpoint of SCD, a primary preventive ICD and sustained VT (HR 1.02, 95% CI 1.00–1.03, *p* = 0.01), independently of other clinical and echocardiographic risk factors
Rodenas-Alesina et al., 2024 [23]	Retrospective cohort; 601 patients with non-ischemic DCM	Composite of VT or VF, appropriate ICD shocks, resuscitated cardiac arrest and SCD	MD ≥ 90 ms, associated with VAs/SCD (HR 2, 7, 95% CI 1.2–6.1, *p* = 0.016), independent of other echocardiographic parameters

## Data Availability

Data sharing is not applicable to this article as no datasets were generated or analyzed during the current study.

## Abbreviations

The following abbreviations are used in this manuscript:

CMR	Cardiac magnetic resonance
DCM	Dilated cardiomyopathy
ECG	Electrocardiogram
GDMT	Guideline-directed medical therapy
HF	Heart Failure
ICD	Implantable cardioverter–defibrillator
LBBB	Left bundle branch block
LGE	Late gadolinium enhancement
LMNA	Lamin A/C gene
LVEF	Left ventricular ejection fraction
MD	Mechanical dispersion
MF	Myocardial fibrosis
NDLVC	Non-dilated LV cardiomyopathy
NT-proBNP	N-terminal pro-B-type natriuretic peptide
SCD	Sudden cardiac death
VAs	Ventricular arrhythmias
